# Disentangling the environmental impact of different human disturbances: a case study on islands

**DOI:** 10.1038/s41598-019-49555-6

**Published:** 2019-09-23

**Authors:** Sebastian Steibl, Christian Laforsch

**Affiliations:** University of Bayreuth and BayCEER, Department Animal Ecology I, Universitaetsstr. 30, D-95440 Bayreuth, Germany

**Keywords:** Urban ecology, Environmental impact

## Abstract

Coastal ecosystems suffer substantially from the worldwide population growth and its increasing land demands. A common approach to investigate anthropogenic disturbance in coastal ecosystems is to compare urbanized areas with unaffected control sites. However, the question remains whether different types of anthropogenic disturbance that are elements of an urbanized area have the same impact on beach ecosystems. By investigating small islands that are utilized for tourism, inhabited by the local population, or remained completely uninhabited, we disentangled different anthropogenic disturbances and analysed their impacts on hermit crabs as indicator species. We observed a negative impact on abundance on tourist islands and a negative impact on body size on local islands. In comparison to the uninhabited reference, both disturbances had an overall negative impact. As both forms of disturbance also impacted the underlying food resource and habitat availability differently, we propose that the findings from our study approach are valid for most obligate beach species in the same system. This demonstrates that in urbanized areas, the coastal ecosystem is not always impacted identically, which emphasizes the importance of considering the particular type of anthropogenic disturbance when planning conservation action in urbanized areas.

## Introduction

Our planet faces an ever increasing number of environmental problems caused by the growth of the human population and its land demands^1^. One ecosystem that suffers substantially from population growth are coasts. Between 50% and 75% of the world’s population live close to coasts^2^, thereby intensifying the anthropogenic impacts on this fragile environment. Globally, sand-dominated beaches comprise 75% of the ice-free coastline^3^ – and in addition to their inherent ecological value, they form a crucial component of the travel and tourism industries worldwide^4^.

Many ecological studies try to identify factors that impact sandy beach ecosystems for the development of conservation measures^5^. Disruption of sand transport by coastal protection structures, sewage pollution, beach nourishment, tourism, beach cleaning, bait collecting and fishing have previously been characterized as anthropogenic disturbances with negative consequences for the beach ecosystem^3^. Under the assumption that these human activities lead to similar ecological consequences and due to the difficulty of a distinct spatial separation of single elements, a common approach to evaluate human disturbances for beach ecosystems is the comparison between urbanized areas and remote, unaffected control sites^6–8^. However, it remains unclear whether various types of anthropogenic disturbances within urbanized areas (e.g. permanent settlements, infrastructure, tourist facilities, etc.) actually have similar impacts on the environment^9^. If not, then current conservation efforts might be improvable by developing strategies that are more specifically tailored to counteract the environmental degradation of the distinct human disturbance.

To investigate this question, the present study was conducted on small coral islands which were either (I) inhabited by the local population, (II) accommodating a tourist facility, or (III) completely uninhabited. This approach guaranteed a distinct spatial separation of two different anthropogenic disturbances and enabled a comparison to ecosystems with no permanent and direct human impact.

A terrestrial hermit crab community comprising two species (*Coenobita rugosus* and *C. perlatus*) was chosen as an indicator to investigate human disturbances^10^. Terrestrial hermit crabs are a crucial component in beach ecosystems that link the marine and the terrestrial food web^11^. As adult terrestrial hermit crabs are restricted to the beaches, populations on small coral islands – like most beach-associated macrofauna – cannot avoid human stressors by migration^10^. Consequently, they can be considered representative of a large number of beach-associated taxa for the purpose of examining anthropogenic disturbances.

## Results

### Impact of different human disturbance on the abundance and size of hermit crabs

**Figure 1:**
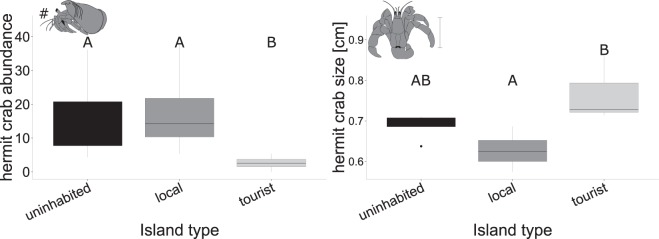
Anthropogenic impact on the abundance and size of hermit crabs. Hermit crab abundance (left) and hermit crab size (right) compared between uninhabited, local and tourist islands (*N* = 4). Significant differences between island types are indicated by different letters.

The studied organisms belonged to the only terrestrial genus of hermit crabs, *Coenobita*, and comprised *C. rugosus* and *C. perlatus*. Significant differences in the abundance and size of the investigated hermit crabs were observed between uninhabited, local and tourist islands (Fig. 1). Island type had a significant effect on the hermit crab abundance within the investigated plots (ANOVA: *N* = 4, *df* = 2, *F* = 28.997, *P* < 0.001). Significantly fewer hermit crabs were present in the plots on tourist islands than on uninhabited (*P* < 0.001) and local islands (*P* < 0.001). The abundance within the plots did not differ between uninhabited (16.25 ± 7.03 mean ± standard error) and local islands (17.87 ± 6.98; *P* = 0.692), although the availability of suitable habitats was significantly reduced on local islands, which might ultimately result in a reduced island population size on the local islands as well (see results section (c)). Furthermore, island type had a significant effect on the hermit crab size (ANOVA: *N* = 4, *df* = 2, *F* = 5.764, *P* = 0.028). On local islands, the investigated hermit crabs were significantly smaller compared to tourist islands (*P* = 0.022). No significant differences were observed between the size of hermit crabs on uninhabited (0.68 ± 0.01 cm) and on local islands (0.62 ± 0.02 cm; *P* = 0.292), nor between uninhabited and tourist islands (0.76 ± 0.04 cm; *P* = 0.201).

**Figure 2:**
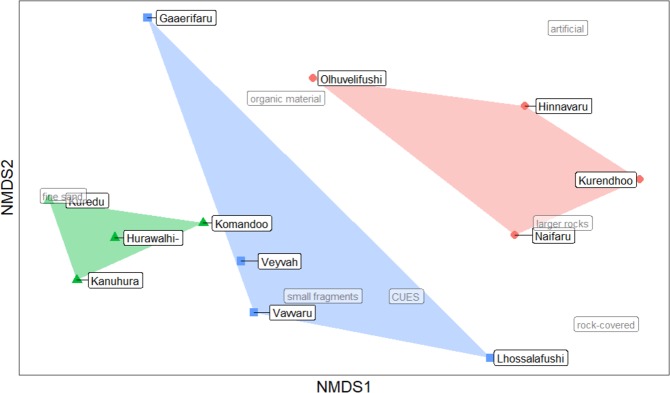
Distinctness of the three investigated island types. NMDS ordination of the investigated islands (blue squares and blue cluster area: uninhabited islands, red circles and red cluster area: local islands, green triangles and green cluster area: tourist islands) is based on the three resource and habitat parameters that influence hermit crab abundance and size (food, shell and habitat availability)). NMDS ordination thereby groups points, i.e. islands, with similar values closer together. Spatial proximity of a data point, i.e. an island, to one of the investigated parameters shows that the island is described by high values in the respective parameter.

To elicit potential reasons for the differences in hermit crab abundance and size between the three island types, food availability, beach habitat structure and empty shell resource were investigated using NMDS (Fig. 2). The three island types differed significantly in resource and habitat (PERMANOVA: *N* = 4*, df* = 2, *F* = 4.770, *P* = 0.004). For a more detailed analysis, each parameter was further investigated specifically.

### Impact of different human disturbances on the food resource of hermit crabs

Island type had no significant effect on the amount of organic material per m² on the beach (Kruskal-Wallis: *N* = 4, *df* = 2, *χ²* = 4.653, *P* = 0.097), but calculated means suggest a non-significant tendency towards fewer organic material on tourist islands (1.14 ± 0.28 g), compared to uninhabited islands (4.63 ± 1.09 g) and local islands (2.85 ± 1.19 g).

### Impact of different human disturbances on the beach habitat structure

**Figure 3:**
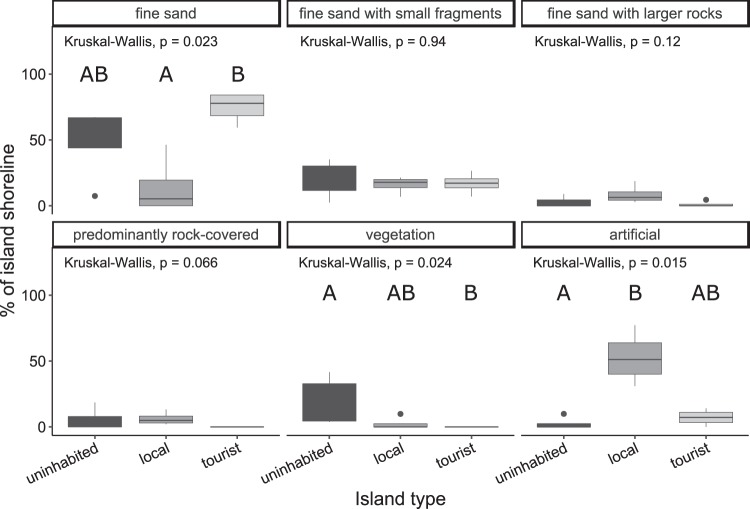
Beach habitat composition of the three island types. Proportions of each of the six categorized beach types on the three investigated island types (*N* = 4). Significant differences in the pairwise comparisons between island types are indicated by different letters.

The composition of the beach habitat (for categorization see methods section and Fig. S1) varied significantly between the three island types (Fig. 3): the proportion of the fine sand beach habitat on the total island’s circumference was significantly different between the three island types (Kruskal-Wallis: *N* = 4, *df* = 2, *χ²* = 7.565, *P* = 0.022), with a significantly higher proportion of fine sand beach on tourist islands than on local islands (*P* = 0.018). Additionally, the proportion of artificial shoreline (Kruskal-Wallis: *N* = 4, *χ²* = 8.459, *P* = 0.014) and vegetation-covered beach (Kruskal-Wallis: *N* = 4, *χ²* = 7.461, *P* = 0.024) was significantly altered, with a significantly higher proportion of artificial shoreline on local islands than on uninhabited islands (*P* = 0.013) and significantly fewer vegetation-covered beach on tourist islands than on uninhabited islands (*P* = 0.026). No significant differences were observed in the proportion of “fine sand with small fragments” habitat (Kruskal-Wallis: *N* = 4, *χ²* = 0.115, *P* = 0.944), “fine sand with larger rock” habitat (Kruskal-Wallis: *N* = 4, *χ²* = 4.832, *P* = 0.089) and “predominantly rock-covered beach” habitat (Kruskal-Wallis: *N* = 4, *χ²* = 5.434, *P* = 0.066). The adjacent shore composition did not differ significantly between the three island types (Kruskal-Wallis: Seagrass: *N* = 4, *χ²* = 0.927, *P* = 0.629, Seagrass and Sand: *N* = 4, *χ²* = 1.457, *P* = 0.483, Sand: *N* = 4, *χ²* = 0.731, *P* = 0.694, Sand and Rock: *N* = 4, *χ²* = 2.457, *P* = 0.293, Rock: *N* = 4, *χ²* = 4.352, *P* = 0.114).

The investigated beach habitat types had a significant effect on the hermit crab abundance (crossed fixed-factor ANOVA island type x habitat type: *N* = 4, *df* = 3, *F* = 5.969, *P* = 0.001), but beach type and island type did not interact significantly (*N* = 4, *df* = 5, *F* = 0.427, *P* = 0.827). When considering the abundance of hermit crabs in only one of the four investigated beach habitat types, island type still had a significant effect on the hermit crab abundance: the abundance of hermit crabs in the “fine sand beach” habitat differed significantly between the three island types (Kruskal-Wallis: *N* = 4, *df* = 2, *χ²* = 15.920, *P* < 0.001), with significantly fewer hermit crabs in the fine sand habitat of tourist islands than in that of uninhabited islands (*P* < 0.001) and of local islands (*P* = 0.035). Island type had also a significant effect on the abundance of hermit crabs in the “fine sand with small fragments beach” habitat (Kruskal-Wallis: *N* = 4, *df* = 2, *χ²* = 12.501, *P* = 0.001) with significantly fewer hermit crabs in this habitat type on tourist islands than in uninhabited islands (*P* = 0.007) and local islands (*P* = 0.007).

### Impact of different human disturbances on the shell resource of hermit crabs

Island type had a significant effect on the overall abundance of empty shells (Kruskal-Wallis: *N* = 4, *df* = 2, *χ²* = 7.130, *P* = 0.028) and on the crab-per-utilizable-empty-shell (CUES)-ratio (Kruskal-Wallis: *N* = 4, *df* = 2, *χ²* = 7.730, *P* = 0.020). This CUES-ratio can be understood as a measure for the intensity of competition over the shell resource. Higher values of this ratio indicate a more severe competition, while values closer to 1 indicate that for each hermit crab a potential utilizable empty shell is readily available. The CUES-ratio was significantly smaller on tourist islands than on uninhabited islands (*P* = 0.024). On uninhabited islands, on average 10 hermit crabs competed over one shell, while on local islands only 6 hermit crabs competed over one shell. For each hermit crab on a tourist island existed on average one utilizable empty shell. Island type had a significant effect on the abundance of non-utilizable empty shells (Kruskal-Wallis: *N* = 4, *df* = 2, *χ²* = 6.545, *P* = 0.037): significantly more non-utilizable empty shells were found on local islands than on uninhabited islands *P* = 0.046) and on tourist islands (*P* = 0.046), while the number of non-utilizable empty shells did not differ statistically between uninhabited and tourist islands (*P* = 0.922). To investigate the reasons for the hermit crab size differences, the shell parameter that most strongly determines hermit crab size, i.e. the aperture area of the shell (Spearman: *R²* = 0.861, *P* < 0.001), was analysed. The aperture area of utilized shells did not differ significantly between the three island types (Kruskal-Wallis: *N* = 4, *df* = 2, *χ²* = 5.303, *P* = 0.070). The aperture area of utilizable empty shells did not differ between the three island types (Kruskal-Wallis: *N* = 4, *df* = 2, *χ²* = 3.803, *P* = 0.149).

## Discussion

Numerous studies have demonstrated that coastal ecosystems are substantially altered or degraded in urbanized areas^6–8^. Due to spatial proximity, different anthropogenic disturbances impact beach ecosystems simultaneously in those areas. It is therefore difficult to disentangle the environmental impacts of different disturbances and investigate with certainty whether ecosystems respond differently to different disturbances^9^. We investigated this issue by studying small coral islands, where different anthropogenic disturbances are spatially separated. The results from our novel study approach show that these disturbances are having clear but distinct impacts on the investigated terrestrial hermit crabs. These findings, based on our study approach, should be transferable to a large number of beach-dwelling taxa, as food and habitat availability generally limit species distribution and population size^12,13^.

On tourist islands, hermit crabs were significantly less abundant and significantly larger than on local islands. Compared to the uninhabited reference system, the abundance was negatively impacted on tourist islands, but did not differ compared to local islands. However, the overall population size on local islands should be considered reduced, as the availability of suitable habitats has been reduced by harbours and coastal protection structures. Therefore, different elements of urbanized areas, i.e. permanent settling or tourism, can have distinct environmental impacts on beach ecosystems.

Food, habitat availability and empty shell abundance are limiting resources for hermit crabs and might offer reasons for the observed differences between the two different land uses^12,14^. The tendency towards less organic material on tourist islands (1.14 ± 0.31 g/m²) compared to local islands (4.26 ± 3.43 g/m²) and the uninhabited reference (4.88 ± 1.84 g/m²) could be explained by beach grooming measurements, which were performed on all four studied resort islands up to four times per day (personal communication). Beach grooming is a common practice around tourist facilities and aims to remove washed-up organic material and debris from the beaches^9^. It causes a reduced food availability for the affected beach fauna, which can result in decreased population densities^15^. In concordance, on average only three hermit crabs per plot were found on the groomed beaches of the tourist islands, compared to 16 hermit crabs on average on uninhabited islands. The beach fauna on the tourist islands might also experience a higher mortality from the cleaning process, either when getting accidentally removed together with the algal material (personal observation) or when being mechanically crushed in the cleaning process, as already demonstrated for ghost crabs^16^. Hence, we hypothesize that beach cleaning is one reason for the significantly decreased abundance on islands with tourist facilities. As beach cleaning was not performed on local islands, hermit crab abundance in suitable habitats remained unaffected (average 18 hermit crabs per plot), although beaches are also used by the local population for recreational activities.

Apart from the overall availability of organic material, the beach habitat structure needs to be considered when investigating the population structure of the beach fauna^17^: compared to the structurally more complex beach habitat types, the fine sand beaches had a significantly reduced hermit crab abundance on all three island types. On tourist islands, this fine sand beach habitat accounted for 75 ± 12% of the total circumference. However, the higher proportion of the more sparsely inhabited fine sand beach cannot be held solely responsible for the reduced hermit crab abundance on tourist islands. Less than one hermit crab per plot was collected in the fine sand beach habitat of tourist islands, while on average eleven hermit crabs were present in the fine sand beach habitat on uninhabited islands. Therefore, disturbances associated with tourist facilities are probably responsible for the reduced abundance on the fine sand beaches of tourist islands. Beach nourishment, a technique where sand gets extracted from the adjacent benthic zone and deposited on the existing shoreline to extend the sandy beaches desired by tourists, is often performed to an extent where the whole natural beach shoreline becomes artificially altered to unvegetated sandy beaches^18^. This measurement can reduce the population size of the whole beach fauna^10,19,20^ – especially when the beach-associated vegetation is completely removed, many beach taxa can become completely absent^10,18^. Therefore, we hypothesize that the removal of beach-associated vegetation, together with the removal of organic material caused by beach grooming and nourishment, are the main drivers for the reduced hermit crab abundance on the islands with tourist facilities.

The shoreline of local islands was differently altered and affected than that of tourist islands: the shoreline of local islands was 53 ± 21% artificially obstructed in form of concrete walls, either for harbour sites or to stabilize reclaimed land. Hence, on average only about half of the local islands shoreline formed a soft-bottom beach habitat suitable for beach-associated organisms^21^. Although the abundance in the investigated plots on local islands were similar to those on uninhabited islands, local islands as a whole, with their extensive artificial shorelines, must be considered as degraded coastal ecosystems with reduced and fragmented beach habitats^22^. In conclusion, this suggests that the total hermit crab population size of a complete local island is on average 50% smaller than the overall population size of uninhabited islands, as the constructions on local islands caused the shoreline to become widely uninhabitable for these organisms^23^. However, the hermit crab abundance within suitable beach habitats did not differ between uninhabited and local islands. This demonstrates that beach-dwelling organisms can occur in densely populated areas in the same high abundance as they do on uninhabited islands, as long as the beach habitat itself remains intact and not altered by human activities.

Besides food availability and habitat structure, shell availability is the most limiting resource for hermit crabs, as they are dependent on the input of empty gastropod shells from the adjacent coastal waters^24^. Therefore, analysing patterns in the shell resource might offer further explanations for the observed differences between the different island types.

The number of non-utilizable empty shells, like cones or cowries, can be considered as a proxy for the overall shell input of an island as these shells accumulate on the beaches without getting removed or utilized by hermit crabs^25^. The number of non-utilizable empty shells did not differ between uninhabited and tourist islands, suggesting that the overall input of the shell resource was similar on both island types. Taken together with the significantly reduced CUES-ratio on tourist islands (on average, one utilizable empty shell per hermit crab was available), neither a diminished shell input, nor high competition over the shell resource, are responsible for the significantly decreased population densities on tourist islands. A sufficient number of empty shells can result in a strong growth of a hermit crab population in a natural system^24^. This suggests that, based on the availability of the shell resource, populations on the tourist islands would have the potential to further grow, but are probably limited due to beach grooming or removal of vegetation.

On local islands however, the number of non-utilizable empty shells was on average four times higher than on uninhabited islands. Harvesting of gastropods for consumption has been shown to provide a surplus of empty gastropod shells for hermit crab populations and might be responsible for the overall increase in shells on local islands^26^. Furthermore, an overall higher gastropod population density in the adjacent coastal waters might be an additional reason for the increased empty shell abundance. This might stem from a greater food supply resulting from wastewater release^27^. This effect only occurred on the local islands, as sewage and other municipal waste is released mostly untreated into the coastal water, while tourist resorts collect the effluents in septic tanks, thereby minimizing nutrient enrichment of the adjacent waters^28^.

The higher abundance of empty gastropod shells on local island beaches is beneficial for the hermit crab populations, as the limiting resource becomes largely available^29^. This is also shown by a decreased CUES-ratio on local islands, suggesting a reduced competition over the shell resource compared to the uninhabited reference. This could explain at least partially why the hermit crab abundance within the investigated plots remained unaffected on the local islands in the present study.

Although the abundance within the investigated plots was not affected negatively, the mean body size on local islands was decreased compared to tourist islands. The body size of a hermit crab correlated with the aperture area of its utilized shell. Therefore, analysing the aperture area of the utilizable empty shells might provide an explanation for the reduced body size on local islands, as the size of the aperture limits growth^30^. However, the aperture areas of both the utilized shells and the utilizable empty shells did not differ significantly between the three island types. This suggests that a lack of larger empty shells is not the main driver for the reduced body size in hermit crabs on local islands, as enough large-sized shells were available, potentially allowing the hermit crabs on the local islands to further grow. Therefore, we hypothesize that human activities on the local islands are responsible for the reduced body size: beach-dwelling decapod crustaceans, like *C. perlatus*, are widely used as fishing bait by the local fishermen^31^. They may select for bigger specimen, as they are easier to find and more suitable as fishing bait^32^. A size-selective harvesting could result in smaller body sizes on local islands, compared to uninhabited and tourist islands, where harvesting is absent^33^. A comparable human-driven size selection is already known in commercial gastropod and fish species, where intensive harvesting and fishing resulted in a shift towards smaller body size due to overexploitation of the larger-sized specimen^34,35^. In comparison, hermit crabs were significantly larger on tourist islands. This can be linked to the reduced abundance on these islands, as a smaller population size decreases intraspecific competition, which ultimately can enable organisms to grow larger^11^.

Our study reveals that two elements of urbanized areas have different environmental impacts. Abundance was negatively impacted on tourist islands, whereas body size was negatively impacted on local islands. Although the abundance within the investigated plots was unaffected on local islands, it is negatively impacted on a larger scale, as about half of the shoreline consists of concrete walls for harbour sites and coastal protection and is therefore uninhabitable for all beach-dwelling organisms.

Here, it is demonstrated that the environment is not always impacted identically by the different elements of an urbanized area, but rather that the type of anthropogenic disturbance is decisive for the ecological consequence. At the same time, organisms can maintain the same population size in densely populated areas as in uninhabited ecosystems, as long as certain habitat characteristics remain unaffected. Our novel approach using small islands thereby ensured that the observed environmental impacts are attributable to only one element of an urbanized area, namely tourism or permanent settlement.

The implications of this study are beneficial for environmental protection measures, as it demonstrates the importance of disentangling various types of disturbance that stem from urbanized areas and to consider each element specifically when developing management strategies for conservation^36^. In practical terms this could mean that the prime measurement for tourist facilities is to reduce beach grooming and leave seagrass and other allochthonous material as a food resource for the beach fauna. The prime measurement for permanently colonized land on the other hand would be to minimize the obstruction of the shoreline by concrete structures and implement some regulations that leave parts of the shoreline as natural sandy beaches. These two proposed management implications to counteract two different forms of land use underline how important it is to disentangle anthropogenic disturbances. A greater understanding of how specific human actions lead to certain environmental responses, will enable us to better curtail these stressors and counteract the global loss of biodiversity and ecosystems^37^.

## Methods

The research was conducted under the permission of the Ministry of Fisheries and Agriculture (Male’, Maldives), permit number: (OTHR)30-D/INDIV/2017/122 and in accordance with the given guidelines and regulations.

Sampling was carried out on 12 small coral islands, all located within the Lhaviyani (Faadhippolhu) Atoll, Republic of Maldives (see Fig. S2). The islands were assigned into three categories: (I) islands that were inhabited solely by the local Maldivian population (local islands), (II) islands with a tourist resort (tourist island) and (III) islands with no permanent direct human disturbance (uninhabited islands) (see Table S1 and Fig. S3). Note that Vavvaru island is strictly speaking not a completely uninhabited island but was a former marine biology field station (Korallionlab). However, the station has closed and during its active time only inhabited three to five staff members and occasionally guest researchers. Sampling of the island’s beaches was carried out from 03/02/2017 to 10/03/2017, always within 2 hours before low tide until low tide. The whole island’s beachline and the adjacent shore were mapped with GPS (eTrex Vista® Cx, Garmin Ltd., Schaffhausen, Switzerland) by assigning it in the following habitat categories: artificial, vegetation-covered (i.e. inaccessible beach, covered fully by shrub vegetation), predominantly rock-covered beach, fine sand with larger rocks, fine sand with small fragments and fine sand beach for the beachline (see Fig. S1) and seagrass, seagrass & sand, sand, sand & rock, rock for the adjacent shore. The percentage of each habitat on the total circumference of each island was calculated.

Each beach was sampled in the abovementioned beach habitat types, distributed randomly over the natural shoreline of the island. The vegetation-covered beach habitat and artificial shorelines were excluded from the sampling due to their inaccessibility. To minimize a biased selection of the sampled part of the beach, the location of the plot was chosen from a distance of minimum 15 m, so that the present hermit crabs could not have been seen in advance. The sampling plots were chosen to guarantee that each present beach habitat type was sampled at least once. Additionally, the two dominant habitat types (i.e. highest percentage of the islands circumference) of every island were sampled in a second plot. When one habitat type was not present on an island or covered less than 10 m in length (i.e. the plot size), an additional plot within the dominant habitat type was sampled, resulting in a total of six plots per island.

Each plot was 10 m long and 2 m wide, measured landwards from the present drift line using a folding rule and a measuring tape. The position of every plot was documented using GPS. All hermit crabs and all empty shells within the plot were counted, collected and stored in a plastic bucket for further analysis.

To assess the amount of potential food, the organic debris in four 0.5 m × 0.5 m sub-plots (resulting in 1 m² per plot in total) within each plot was collected using forceps and stored in a plastic bag. The four sub-plots were positioned at equal distances in a diagonal manner within the plot (0 m, 3.3 m, 6.6 m and 10 m along the plot length and at distances of 1.5 m, 1.0 m, 0.5 m and 0 m from the drift line; Fig. S4). The wet weight of the organic material per plot was measured using a fine scale (TS-300 300 g × 0.01 g, G&G GmbH, Neuss, Germany).

Hermit crabs were removed from their shell by carefully heating the apex of the shell above an open flame. This is a standard procedure to remove hermit crabs from their shells and leaves the animal without injuries^38,39^. Hermit crabs were photographed on millimetre paper (Nikon D5000 mounted with Nikon AF-S Nikkor 18–105 mm, 1:3.5–5.6, Nikon Corp., Tokyo, Japan).

All shells (utilized and empty) were photographed on millimetre paper and identified using morphological identification keys^40–43^. All empty shells were assigned in two categories: (I) empty shells belonging to a gastropod species that was found to be utilized by a hermit crab and therefore considered being in general utilizable, and (II) empty shells belonging to a gastropod species, which was never found to be utilized by a hermit crab (mainly cone or cowrie shells) and therefore considered to be generally not utilizable by the investigated hermit crab species. Non-utilizable empty shells, like cowrie or cone shells, accumulate on the beaches without being ever utilized or transferred over longer distances by hermit crabs or any other beach inhabitant^25,44^ and can therefore be used as a proxy for the overall shell input on the beaches.

After this procedure, the hermit crabs were transferred into a plastic bucket together with their removed shell and left to recover before being transferred back to their original beach habitat.

The size of the hermit crabs and their corresponding shell was determined using ImageJ 1.49b (Rasband, W.S., ImageJ, U. S. National Institutes of Health, Bethesda, Maryland, USA, http://imagej.nih.gov/ij/, 1997–2015) by measuring the carapace length of the hermit crab, and the length and width of the aperture area of each shell.

The statistical analysis was carried out using R 3.5.1, extended with the “vegan” package for multivariate ecological analysis^45^. Prior to statistical analysis, abundance data was Tukey-transformed (lambda = 0.375) to meet the assumptions of normality and variance homogeneity. Where assumptions for parametric testing were violated, non-parametric Kruskal-Wallis tests were conducted. To test for differences in hermit crab abundance between the three island types (uninhabited, local, tourist islands) and account for the different habitat types on each island, univariate ANOVA with crossed fixed factors (island type x habitat type) was performed and pairwise comparisons were calculated using TukeyHSD post-hoc tests (*N* = 4). The influence of human land use on hermit crab size was analysed by calculating the mean body size for each island and statistically compare it between the three island types (*N* = 4) using ANOVA and TukeyHSD post-hoc tests. To investigate how the two different forms of human land use influence the underlying resources of hermit crabs, a non-metric multidimensional scaling (NMDS) was performed. First, the parameters “empty shell abundance”, “organic material” and the proportion of the four different beach habitat types were rescaled between 0 and 1 for Bray-Curtis dissimilarity matrix calculation. Then, NMDS ordination was calculated using *k* = 2 dimensions. To test for differences in resource availability between the three island types based on the NMDS, a PERMANOVA was calculated (Bray-Curtis, 4999 permutations). Additionally, Kruskal-Wallis tests with Dunn post-hoc tests and Bonferroni corrections were performed to compare the underlying resources (i.e. organic material [g/m²], empty shell abundance, and proportion of each beach habitat type) separately between the three island types (*N* = 4). The abundance of hermit crabs within the “fine sand with larger rocks”- and the “predominantly rock-covered”-beach habitat were not compared individually between the three island types, as the “fine sand with larger rocks”-habitat occurred only on 50% of all investigated islands and the predominantly rock-covered beach was overall absent on tourist islands. To further investigate reasons for the differences in hermit crab size between the three island types, the shell parameter that correlated best with hermit crab size was identified using Spearman rank correlation test. The aperture area of the shell showed a high correlation with hermit crab body size (*R²* = 0.861, *P* < 0.001) and was subsequently compared for utilized and utilizable empty shells between the three island types using Kruskal-Wallis tests.

The datasets generated during this study are available from the corresponding author on reasonable request.

## Supplementary information

supplementary info
