# Supplementary material for: Disentangling the environmental impact of different human disturbances: a case study on islands
Source: Sci Rep. 2019 Sep 23;9:13712. doi: 10.1038/s41598-019-49555-6 (PMC6757039; doi:10.1038/s41598-019-49555-6)
Supplement: Supplementary file 1 — supplementary info [file 41598_2019_49555_MOESM1_ESM.docx]

Supplementary Information for

“Disentangling the environmental impact of different human disturbances: A case study on islands”

**Authors**

Sebastian Steibl ([sebastian.steibl@uni-bayreuth.de](mailto:sebastian.steibl@uni-bayreuth.de))

Christian Laforsch^*^ ([christian.laforsch@uni-bayreuth.de](mailto:christian.laforsch@uni-bayreuth.de))

*: Corresponding author


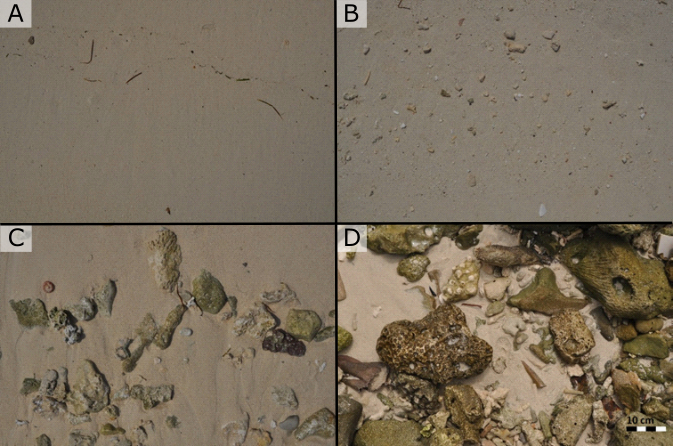


**Figure S1:** Categorized types of beaches: (1) fine sand, (2) fine sand with small fragments (3) fine sand with larger rocks, (4) predominantly rock-covered.


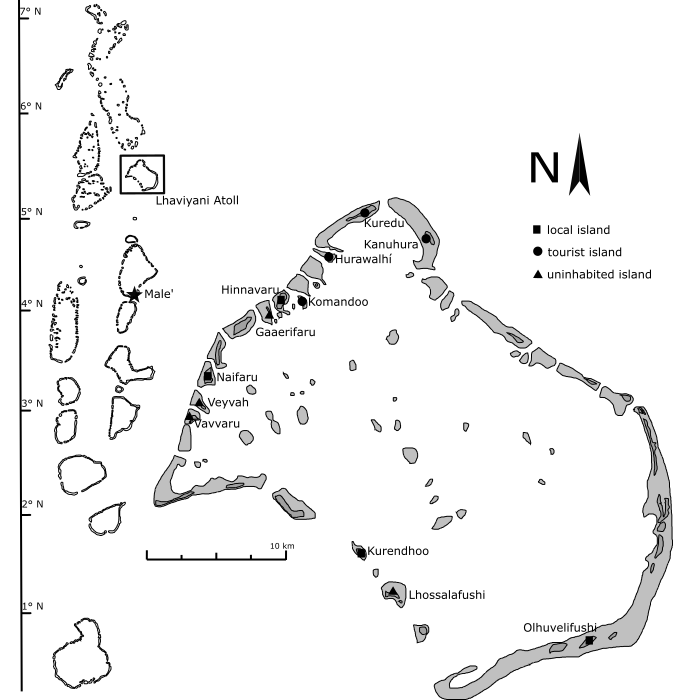


Figure S2: Location of the Lhaviyani Atoll within the Republic of Maldives shown on the left side. Detail view of the Lhaviyani Atoll with all sampled islands is shown on the right. Local islands are indicated with a square, tourist islands with a circle and uninhabited islands with a triangle (see also Table S1).

**
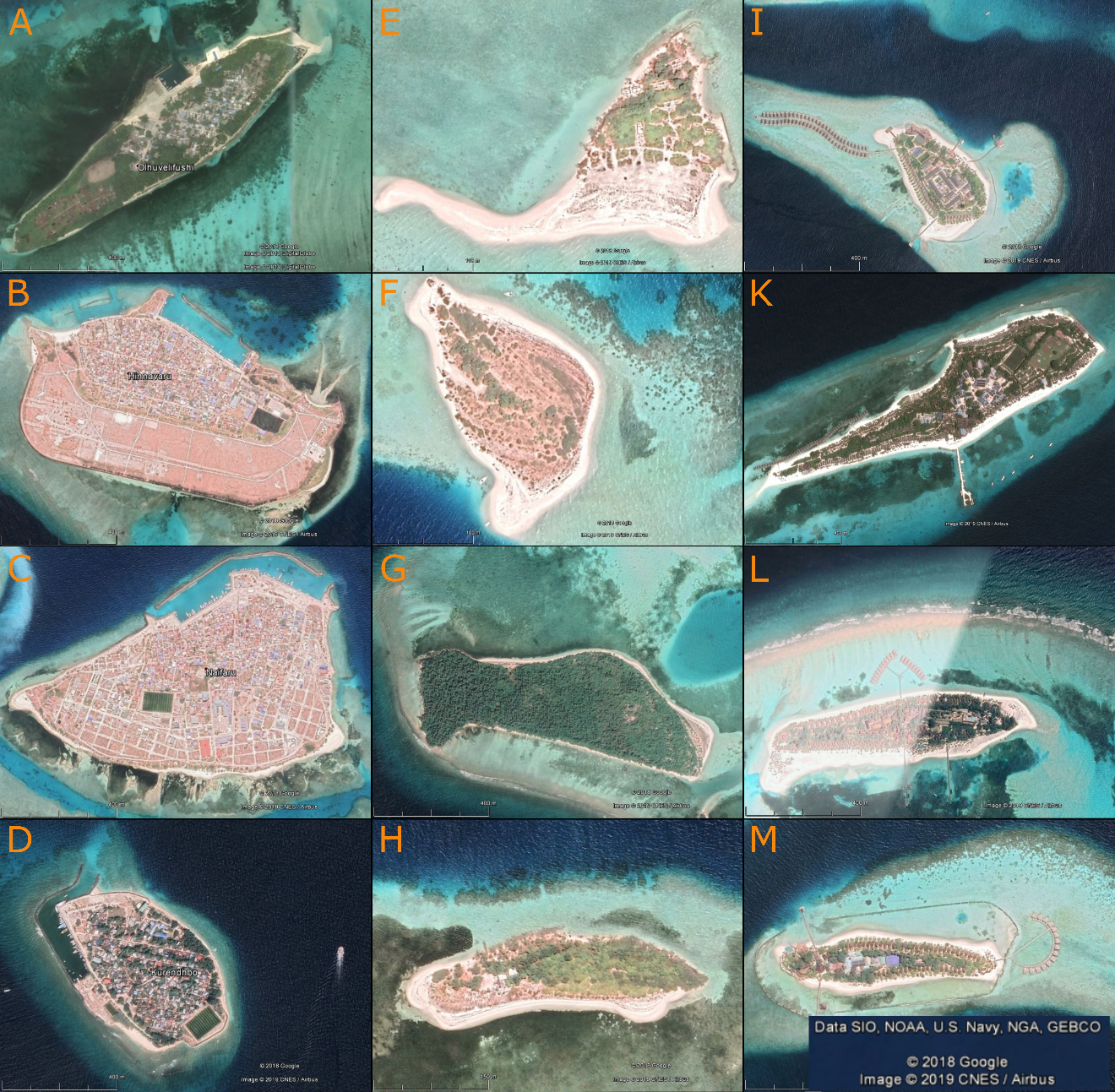
**

**Figure S3:** Satellite images (obtained from Google Earth 7.1.5.1557; Map data: Google, CNES / Airbus 2019) of the twelve investigated islands: A-D (left column) local islands, E-H (central column) uninhabited islands, I-M (right column) tourist islands. Note that islands are not to scale. Local islands (A-D) are overall characterized by a high degree of impervious surface (such as roads, houses, harbour, etc.) and a reduced vegetation cover, except for A, where the western side of the local island was agricultural land. Tourist islands (I-M) also have housing sites (guest bungalows, restaurants, swimming pools etc.), but at the same time an overall higher degree of intact vegetation.


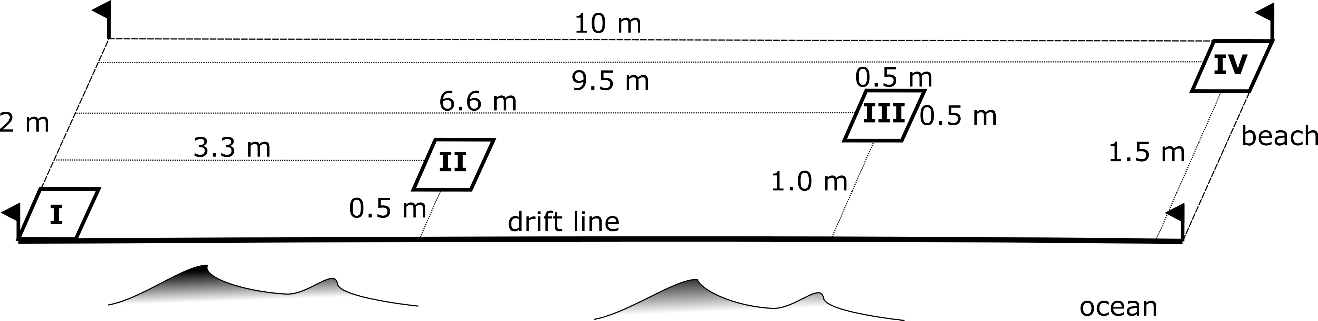


**Figure S4:** Schematic illustration of the sampling procedure. Each plot measured 10x2 m and was positioned along the drift line. Within each plot, all hermit crabs and empty shells were collected. To assess the amount of washed-up organic material, four sub-plots (I-IV), measuring 0.5x0.5 m each, were positioned at 0 m, 3.3 m, 6.6 m, and 9.5 m along the shoreline and 0 m, 0.5 m, 1.0 m and 1.5 m perpendicular to the shoreline.

Table S1: Name, type and circumference of the sampled islands, as well as the dates when sampling was conducted. All islands are located in the Lhaviyani Atoll, Republic of Maldives (see Figure S2).

| **Island type** | **Island name** | **Circumference** | **Sampling date, time of low tide** |
| --- | --- | --- | --- |
| Tourist island | Kuredu | 3616 m | 04/02/2017 11:04 |
|  | Hurawalhí | 1186 m | 17/02/2017 09:59 |
|  | Kanuhura | 2182 m | 09/03/2017 16:12 |
|  | Komandoo | 1072 m | 05/03/2017 10:24 |
| Local island | Naifaru | 3312 m | 03/02/2017 10:18 |
|  | Hinnavaru | 3277 m | 19/02/2017 11:06 |
|  | Olhuvelifushi | 2913 m | 08/03/2017 14:42 |
|  | Kurendhoo | 1888 m | 07/03/2017 12:44 |
| Uninhabited island | Veyvah | 775 m | 18/02/2017 10:30 |
|  | Vavvaru | 922 m | 06/02/2017 13:22 |
|  | Gaaerifaru | 853 m | 06/03/2017 11:19 |
|  | Lhossalafushi | 2480 m | 10/03/2017 17:09 |
